# Pacific decadal oscillation causes fewer near-equatorial cyclones in the North Indian Ocean

**DOI:** 10.1038/s41467-023-40642-x

**Published:** 2023-08-28

**Authors:** Shinto Roose, R. S. Ajayamohan, Pallav Ray, Shang-Ping Xie, C. T. Sabeerali, M. Mohapatra, S. Taraphdar, K. Mohanakumar, M. Rajeevan

**Affiliations:** 1 Arabian Center for Climate and Environmental Sciences, New York University Abu Dhabi, Abu Dhabi, UAE; 2 Department of Civil Engineering, McGill University, Montreal, Canada; 3 Department of Meteorology, Abu Dhabi Polytechnic, Institute of Applied Technology, Abu Dhabi, UAE; 4 Meteorology, Florida Institute of Technology, Melbourne, FL USA; 5 Scripps Institute of Oceanography, University of California San Diego, San Diego, CA USA; 6 Climate Research and Services, India Meteorological Department, Pune, India; 7 India Meteorological Department, New Delhi, India; 8 Atmospheric Science & Global Change Division, Pacific Northwest National Laboratory, Richland, WA USA; 9 Advanced Centre for Atmospheric Radar Research, Cochin University of Science and Technology, Kochi, India; 10 Ministry of Earth Sciences, New Delhi, India

**Keywords:** Climate sciences, Atmospheric dynamics

## Abstract

Tropical cyclones do not form easily near the equator but can intensify rapidly, leaving little time for preparation. We investigate the number of near-equatorial (originating between 5°N and 11°N) tropical cyclones over the north Indian Ocean during post-monsoon season (October to December) over the past 60 years. The study reveals a marked 43% decline in the number of such cyclones in recent decades (1981–2010) compared to earlier (1951–1980). Here, we show this decline in tropical cyclone frequency is primarily due to the weakened low-level vorticity modulated by the Pacific Decadal Oscillation (PDO) and increased vertical wind shear. In the presence of low-latitude basin-wide warming and a favorable phase of the PDO, both the intensity and frequency of such cyclones are expected to increase. Such dramatic and unique changes in tropical cyclonic activity due to the interplay between natural variability and climate change call for appropriate planning and mitigation strategies.

## Abstract (web summary)

The north Indian Ocean is a hotbed for Low Latitude Cyclones (LLCs; originating between 5°N and 11°N). This study finds a remarkable decline in the frequency of LLCs in recent decades modulated by the remote influence of Pacific Decadal Oscillation.

## Introduction

Tropical cyclones (TCs) are far fewer in the vicinity of the equator because of the small Coriolis force that cannot provide the initial spin-up of the cyclonic vortex^1^. On average, there are fewer than two cyclones per year within 5° latitude of the equator, with the majority originating in the Western Pacific Ocean^2^. A little away from the equator, however, TCs can form more easily in the presence of a larger Coriolis force and other favorable environmental conditions. These low-latitude cyclones (LLCs, originating between 5° and 11° latitude) are much smaller in size than those in higher latitudes but intensify more rapidly^3–5^ as the boundary layer inflow closer to the cyclonic center is higher in the presence of smaller Coriolis force (smaller inertial instability). The strong boundary layer inflow enhances diabatic heating rates^5^ that induce stronger secondary circulation leading to enhanced boundary layer moisture convergence^6–8^ at low latitudes. This positive feedback mechanism spins up the system more rapidly at low latitudes.

The north Indian Ocean (NIO) in the post-monsoon season (October–November–December or OND) is a hotbed for LLCs that constitute about 60% of all TCs formed in the NIO (since 1951)^9^ but has received relatively less attention. The rapid intensification of LLCs leads to devastating damages due to insufficient warning and preparation time. For example, LLC Ockhi traveled over 2000 Km and devastated parts of Sri Lanka and India with extensive damage to properties and the loss of lives of 884 people in November 2017^10^. Such devastating impact from the NIO LLCs motivates us to study the variability of the LLC with the available data. Interestingly, the Indian Ocean basin has warmed consistently and more than any other ocean basin^11–13^. Since the genesis of tropical cyclones is closely linked with the underlying sea surface temperature (SST)^1,14^, a study on the association of trends in the SST with LLC frequency assumes significance.

In this work, we show that LLC frequency over the NIO during the post-monsoon season has declined by 43% in recent decades (1981–2010) compared to earlier (1951–1980). This decline in LLC frequency is primarily due to the weakened low-level vorticity modulated by the Pacific Decadal Oscillation (PDO) and increased vertical wind shear. We also show that this influence of PDO on LLC frequency is largely independent of El Niño Southern Oscillation (ENSO) influence.

## Results

### Epochal changes in the LLCs in the Bay of Bengal

**Fig. 1:**
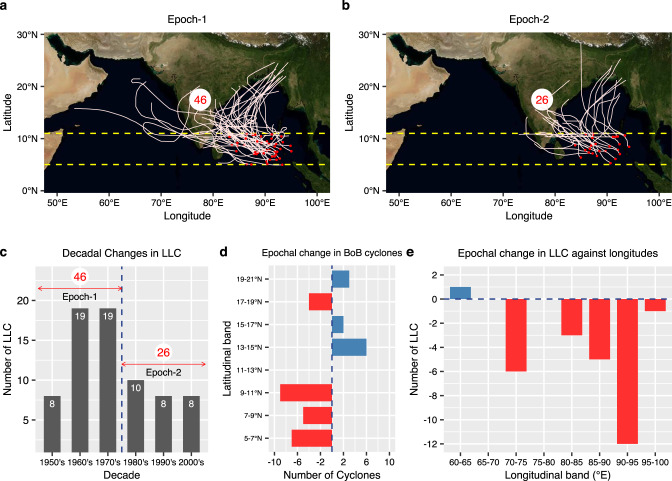
Epochal change in low-latitude cyclone (LLC) frequency in the Bay of Bengal (BoB). **a** The number of LLCs and their tracks over the BoB (83°–95°E, 5°–11°N) in the post-monsoon seasons (October–November–December) during epoch-1 (1951–1980) and (**b**) epoch-2 (1981–2010). The dashed lines denote the low-latitudinal belt (5°–11°N). The red labels represent the total number of LLCs formed over 83°–95° E, 5°–11° N. **c** Decadal variation in the number of LLCs formed over BoB (formed over 83°–95°E, 5°–11°N). **d** Latitudinal distribution of the epochal difference in the number of cyclones in the BoB (80°–100°E). **e** Longitudinal distribution of the epochal difference in the number of LLCs (5°–11°N). Cyclonic storms (>34 knots) and severe cyclonic storms (>48 knots) are considered in this study^9^.

Based on the categorization of TCs (Supplementary Table 1), 72 LLCs formed over the Bay of Bengal (BoB; 83°E–95°E) in the last sixty years (1951–2010), which constitute about 75% of the total NIO LLCs^9,15^. We define two 30-year epochs, namely, epoch-1 (1951–1980, Fig. 1a) and epoch-2 (1981–2010, Fig. 1b) to study the post-monsoonal LLC activity. The decline in the genesis of LLC over the BoB is prominent as only 26 TCs (121 TC days) were formed in the epoch-2 compared to 46 (290 TC days) in the epoch-1, indicating a ≈ 40% decline in the LLC frequency (≈60% decline in the TC days; Fig. 1). This decline in LLC frequency is evident over all three months of the post-monsoonal season over the BoB (Supplementary Fig. 1). Moreover, the TC day density^16^ also declined (Supplementary Fig. 2). The epochal change in LLC frequency arises primarily from the genesis of a record number of 19 LLCs formed during the sixties (1961–1970) and seventies (1971–1980), while other decades report 8–10 LLCs (Fig. 1c). In the entire BoB (80°E–100°E), the number of LLCs (5°N–11°N) declined by 21, whereas the number of cyclones that formed north of 11°N increased by 7 (Fig. 1d). Therefore, for the entire BoB, the increase in TC frequency north of 11°N was only about 33% compared to the decrease in LLC frequency. Considering the 83°–95°E longitudinal band in the BoB where most TCs form, the frequency in LLC (5°–11°N) declined by 20 from epoch-1 to epoch-2 (Fig. 1c), whereas the number of TCs north of 11°N increased by 1 only. Therefore, this decline in LLC in epoch-2 compared to epoch-1 does not represent a meridional shift^17^; rather, it shows an epochal change in the BoB LLC frequency. Furthermore, the 43% epochal decline (46 to 26) in LLC frequency in the BoB is statistically significant, whereas the epochal increase (20–21) in TCs formed north of 11°N is not statistically significant (Supplementary Fig. 3). In the entire NIO (BoB and Arabian Sea), there was a decline in LLC frequency by 27, whereas the number of TCs north of 11°N increased by 12. Therefore, for the entire north Indian Ocean, the increase in TC frequency north of 11°N was about 44% compared to the decrease in LLC frequency (Supplementary Fig. 4). These results are summarized in Supplementary Table 2.

The frequency of LLC in the pre-monsoon season (April-May) is much smaller than that in the post-monsoon season, but a decline in the frequency of LLC is also evident in the pre-monsoon season (Supplementary Fig. 5, Supplementary Table 3). Other available TC datasets over the NIO basin also show a similar decline (Supplementary Table 4). Here, we aim to address the causes behind the remarkable decline in LLC frequency in BoB in the post-monsoon season, specifically its dependence on large-scale environmental factors during 1951–2010. The erudition obtained from such an investigation may aid in enhancing the understanding and prediction of LLC over the NIO.

### Epochal changes in the factors that control LLCs in the BoB

**Fig. 2:**
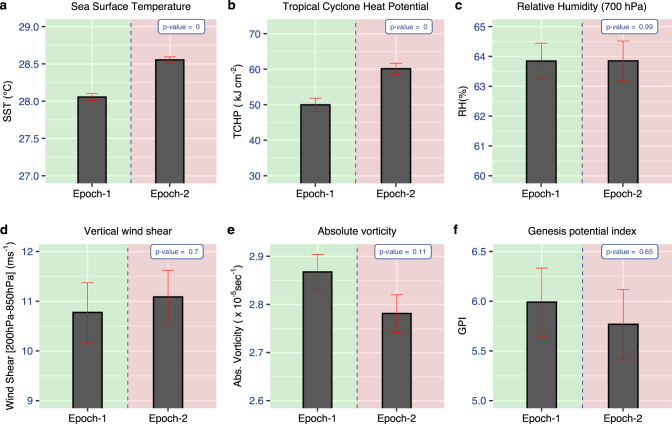
Epochal mean of environmental parameters for tropical cyclone genesis. Epochal mean of (**a**) sea surface temperature (°C) (**b**) tropical cyclone heat potential (KJcm^−2^), (**c**) mid-tropospheric (700 hPa) relative humidity (%), (**d**) vertical wind shear between 850 and 200 hPa (m s^−1^), (**e**) Low-level (925hPa) absolute vorticity (x10^−5^s^−1^), and (**f**) genesis potential index. All are averaged over 5°–11°N, 83°–95°E during post-monsoon season. The standard error (SE, ${{{{{\rm{standard}}}}}}\,{{{{{\rm{deviation}}}}}}/\sqrt{{{{{{\rm{sample}}}}}}\,{{{{{\rm{size}}}}}}}$) is marked as red bars.

The major factors responsible for the genesis of TCs are SST, mid-tropospheric humidity, vertical shear of the horizontal winds, and absolute vorticity^1,18^. Consistent with the warming trend in the Indian Ocean^12,13^, a warming of 0.5 °C is found from epoch-1 to epoch-2 over the low latitudinal belt of the BoB (Fig. 2a). The warming SST, which is much above the SST threshold (26 °C) for cyclogenesis^19^, is expected to support an increase in frequency and intensity of TCs^20–25^, yet the number of BoB LLCs has decreased (Fig. 1). The tropical cyclone heat potential (TCHP), another vital parameter concerning the intensification of TCs^26^, also shows an increasing trend over BoB during 1981–2010 (Fig. 2b) similar to the global oceans^27^. Therefore, a decline in the BoB LLC frequency cannot be attributed to an increase in TCHP. All the above oceanic (SST, TCHP) parameters are working synergistically to create a conducive environment for TC genesis.

**Fig. 3:**
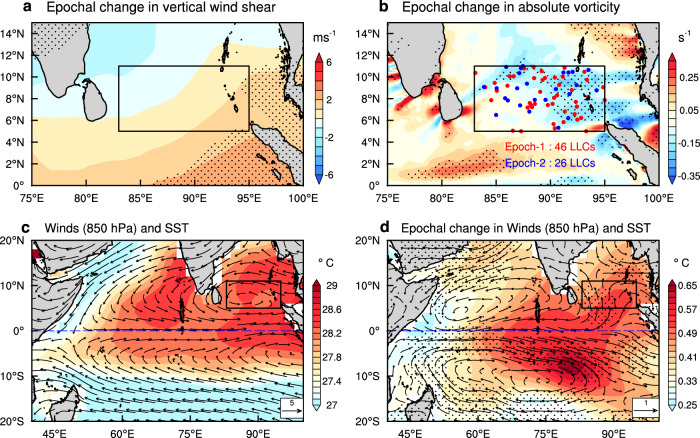
Epochal change in environmental parameters for tropical cyclone genesis. Epochal change (epoch-2 minus epoch-1) in (**a**) vertical wind shear between 850 and 200 hPa (m s^−1^) and **b** 925 hPa absolute vorticity (×10^−5^ s^−1^) during post-monsoon season. Stippled areas in (**a**) and (**b**) represent regions where epochal differences are significant at the 90% confidence level by a Student’s *t* test. Genesis locations of low-latitude cyclones are marked as dots in red (epoch-1) and blue (epoch-2). **c** Post-monsoon seasonal mean winds (m s^−1^, vector) at 850 hPa, and sea surface temperature (SST; °C, shaded) in the tropical Indian Ocean (1951–2010). **d** Epochal difference (epoch-2 minus epoch-1) in winds at 850 hPa and SST. Shaded contours and stippled areas in (**d**) represent regions where epochal differences in SST and winds are significant at the 90% confidence level by a Student’s *t* test, respectively.

The mid-tropospheric relative humidity does not show any epochal variation (Fig. 2c) and hence cannot aid a reduction in LLC frequency. Another important circulation feature that influences cyclogenesis and its intensity is the vertical shear of the horizontal winds, which allows the ventilation of mid-level moisture out of the inner core of the cyclone^28^. As a result, the vertical shear of the intensifying TCs is lower than that of the non-intensifying TCs^28^. Vertical wind shear values greater than 11m s^−1^ are often considered a barrier to TC development^29^. The epoch-2 values of the mean vertical wind shear are above 11m s^−1^ (Fig. 2d), which may limit LLC development. However, vertical shear increased only slightly (≈0.3m s^−1^) during 1981–2010 (Fig. 2d) and the changes are not significant in the core LLC genesis region (83°–95°E, Fig. 3a). Due to these two reasons, it is unlikely that the epochal change in mean vertical wind shear alone will support a significant epochal decline in the BoB LLC frequency. Moreover, this epochal increase in vertical wind shear is not seen in another dataset (Supplementary Fig. 6). The spatial distribution of SST and RH shows an increase in epoch-2 compared to epoch-1 over the LLC genesis region (Supplementary Fig. 7) and hence cannot support an LLC decline. The most significant factor that seems to support the decline in LLC frequency in epoch-2 is the low-level absolute vorticity, which has reduced substantially (Fig. 2e). The reduced absolute vorticity during epoch-2 (Fig. 3b) seems to be responsible for a fewer number of LLCs, especially over 89°E–95°E region (Supplementary Fig. 8), where the changes in absolute vorticity are the largest (Fig. 3b). The dynamics behind the epochal variation of low-level vorticity in the low latitudes and its association with the low-level winds and LLC are examined further. Using an empirically derived index named Genesis Potential Index (GPI^30^, Supplementary Eq. (1)), which provides an integrated measure for cyclogenesis potential, we show that the epochal decline in GPI (Fig. 2f) is in agreement with the observed decline in LLC frequency (Figs. 1c,  3b, Supplementary Fig. 9) and is largely contributed by the decline in absolute vorticity with some influence from vertical wind shear (See Supplementary Eq. (2), Supplementary Table 5).

### Mechanism of epochal decline in low-level vorticity

The low-level winds over the equatorial Indian Ocean are dominated by westerlies during pre- and post-monsoon seasons^31,32^. These low-level equatorial westerlies in the presence of easterlies a little away (≈10°) from the equator lead to the formation of cyclonic circulation on either side of the equator, one between 5°N–11°N and another between 5°S–11°S (Fig. 3c). The difference in wind patterns between the two epochs shows the strengthening of equatorial westerlies in epoch-2 over 0°–5°S (Fig. 3d, vector). This strengthening of westerlies results in the formation of a cyclonic gyre north of the peak westerly winds in the equatorial region (0°–5°N, Fig. 3d). This shift of the cyclonic gyres towards the equator can no longer support cyclogenesis as the Coriolis force is negligible close to the equator. This insufficient background rotation is largely responsible for the reduction in cyclogenesis in the recent epoch over this region.

**Fig. 4:**
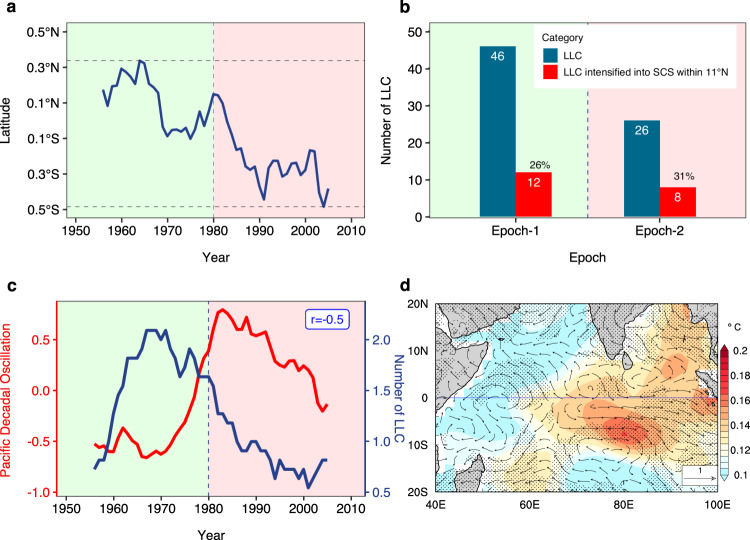
Low-Latitude Cyclones (LLC)-Pacific Decadal Oscillation (PDO) connection and epochal change in LLC intensity. **a** Latitudinal position of zero absolute vorticity (850 hPa) at 80°E. **b** Epochal change in intensities in different categories of LLC. **c** Smoothed PDO index (red) and the number of LLCs (blue) in the Bay of Bengal (83°–95°E, 5°–11°N). The vertical dashed lines separate the two epochs. The correlation between two time series is denoted as r in (**c**). **d** Regressions of the smoothed PDO index onto Sea Surface Temperature (SST, shaded, °C) and 850 hPa winds (vector, m s^−1^) for the period 1951–2010. All calculations were performed for the post-monsoon (October-November-December) season, except for PDO. A 11-year running mean is used to smooth the time series in (**a**, **c**, **d**) to isolate the decadal signal. Shaded contours and stippled areas in (**d**) represent regions where epochal differences in SST and winds are significant at the 90% confidence level by a Student’s *t* test.

The latitudinal shift in the equatorial westerlies and its association with the epochal changes in LLC is further investigated by estimating the changes in the latitudinal position of zero absolute vorticity^33^ (*η* = 0; Fig. 4a). The southward shift in the zero absolute vorticity by ≈0.75°N in thirty years (1960–1990) complies with the decline in the number of LLCs (Fig. 4b). This shift of the cyclonic gyres towards the equator leads to 2 TCs in epoch-2 compared to no TCs in epoch-1 within 0°–3°N^34,35^, but causes a much larger decrease in TC frequency in 5°N–11°N due to reduced vorticity. In epoch-1 12 out of 46 TCs intensified into severe cyclonic storms (SCS; above 48 Knots) before crossing 11°, while 8 out of 26 TCs became SCSs in epoch-2 (Fig. 4b). This represents a marginal increase of TCs to attain higher intensity in epoch-2 compared to epoch-1.

### Connection between epochal decline in vorticity and the PDO

The apparent epochal variation in lower-tropospheric absolute vorticity (Fig. 2e) and its latitudinal shift (Fig. 4a) motivate us to explore whether they are related to the PDO, which is the dominant climate variability in the decadal timescales^36–38^. It is found that the positive phase of the PDO coincides with the epochal decline of LLC during the post-monsoon season (Fig. 4c). The association of PDO phases and LLC frequency leads to the conjecture that the latitudinal shift in westerlies is strongly associated with the PDO phases. To test this conjecture, a regression analysis of the decadal signal of SST and low-level winds with the PDO index is carried out. The resultant pattern (Fig. 4d) is similar to the pattern seen in Fig. 3d with peak SST anomalies around 80°E and 7°S. This provides further evidence that the epochal difference between the SST and the low-level winds is associated with the PDO phases through the latitudinal shift in equatorial westerlies (Fig. 3d). The tropical-wide view of the difference in velocity potential and the associated divergent winds shows an anomalous low-level convergence over the central tropical Pacific and Indian Ocean in response to the cold-to-warm phase transition of PDO from epoch-1 to epoch-2 (Supplementary Fig. 10). The core of the anomalous convergence in the Indian Ocean is south of the equator (Supplementary Fig. 10a). The anomalous divergence over the central Pacific and Indian oceans are more intense in the upper level (Supplementary Fig. 10b). The Coriolis force acting on the southward ageostrophic flow induces the anomalous westerlies (easterlies) south (north) of the equator. This complies with the change in the strength of the Walker circulation associated with the PDO phases. The epochal changes in the SST and the low-level winds (Fig. 4d) are associated with the PDO phases, leading to the southward shift in equatorial westerlies (Fig. 3d). To what extent this LLC-PDO relationship is influenced by the different phases of the ENSO is investigated next.

The role of ENSO phases on PDO and its effect on tropical cyclone frequency is discussed in numerous studies^38–40^. These studies imply that the PDO modulates the climate patterns resulting from ENSO. The teleconnection signal of ENSO can amplify (weaken) in positive (negative) phases of PDO. This does not mean that the PDO physically controls ENSO but rather that the resulting climate patterns interact with each other. Here, irrespective of the ENSO phases, the epochal decline in LLC is prominent in the BoB (Table 1). For example, during El Niño years (9 years in epoch-1 and 10 years in epoch-2), the average number of LLCs per year declined from 1.67 to 0.6. During La Niña years (7 years in epoch-1 and 8 years in epoch-2), the average number of LLCs per year declined from 1.29 to 1. Moreover, out of a total decline of 20 LLCs in epoch-2 compared to epoch-1 (Fig. 1c), 9 declined during El Niño years, 1 declined during La Niña years, and 10 declined during neutral years. This decline in LLC frequency during neutral years shows that the influence of PDO phases on LLC frequency is independent of ENSO influence. However, the BoB LLC decline is prominent during positive ENSO and PDO phases. Interestingly, the ENSO-LLC relationship shows distinct behaviors in two epochs (Table 1). During epoch-2 (1981–2010), there were fewer LLCs during El Niño years (0.6 per year) and higher LLCs during La Niña years (1 per year). This relationship is consistent with Roose et al.^41^, where they found using data from 1979–2020 that the El Niño years have fewer BoB LLCs in the presence of anomalous equatorial easterlies that are not conducive for initiating cyclonic vorticity. On the other hand, La Niña years have a higher number of BoB LLCs in the presence of anomalous equatorial westerlies that are conducive for generating cyclonic vorticity^41^. However, such a relationship between the BoB LLC frequency and ENSO phases is not evident in epoch-1 since there are more LLCs during El Niño (1.67 per year) and neutral (1.57 per year) years compared to La Niña (1.29 per year) years.

**Table 1:** Low Latitude Cyclone (LLC) frequency in different El Niño Southern Oscillation (ENSO) phases

ENSO Phases	Epoch-1 (1951–1980)	Epoch-2 (1981–2010)
El Niño	1.67 (15 LLCs/9yrs)	0.6 (6 LLCs/10yrs)
La Niña	1.29 (9LLCs/7yrs)	1 (8 LLCs/8yrs)
Neutral	1.57 (22 LLCs/14yrs)	1 (12 LLCs/12yrs)

The average number of LLCs (formed between 5°N–11°N) in the Bay of Bengal during the post-monsoon season (October-November-December) during different phases of ENSO.

**Fig. 5:**
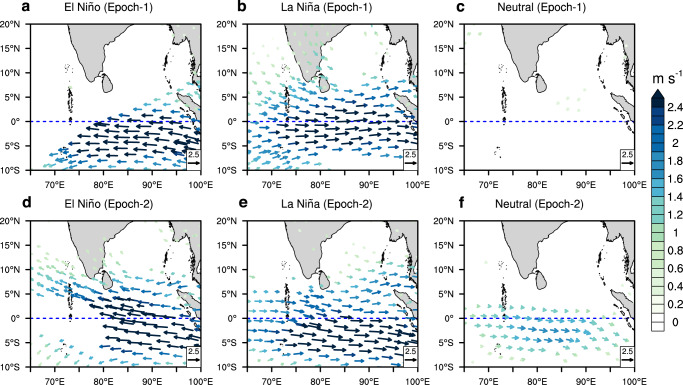
Composite of 850hPa wind anomalies in different El Niño Southern Oscillation phases. Composite of 850hPa wind anomalies (m s^−1^). **a** El Niño (epoch-1), (**b**) La Niña (epoch-1), (**c**) Neutral-ENSO (epoch-1), (**d**) El Niño (epoch-2), (**e**) La Niña (epoch-2) and Neutral-ENSO (epoch-2) years. Only statistically significant (95% confidence level using Student’s *t* test) anomalies are shown.

Further analyses were conducted to understand the underlying causes behind this different LLC-ENSO relationship in two epochs. In epoch-1, the El Niño-induced easterly wind anomaly is displaced in the south of the equator (Fig. 5a). Therefore, the climatological westerly winds north of the equator (Fig. 3c) are less affected by the El Niño-induced anomalies (Fig. 5a), and thereby do not inhibit the initial spin-up for LLC. On the other hand, due to the southward shift in the equatorial westerly winds in epoch-2, the El Niño-induced easterly anomalies increased in the north of the equator (Fig. 5d), which in turn reduced the vorticity in the LLC domain. This causes the LLC frequency to decline in El Niño years in epoch-2 (0.6 per year) compared to epoch-1 (1.67 per year). The epochal change in LLC frequency during La Niña years (1.29 per year in epoch-1 versus 1 per year in epoch-2) is small due to similar wind patterns in both epochs (Fig. 5b, e). The La Niña-induced westerly anomalies are symmetric about the equator in both epochs, and they could generate strong absolute vorticity in the LLC domain (Fig. 5b). However, the La Niña-induced westerly anomaly is shifted southward in epoch-2 (Fig. 5e), which could generate less absolute vorticity in the LLC domain than in epoch-1. Moreover, the anomalous westerly winds in the south equatorial Indian Ocean are also evident in the Neutral-ENSO composites during epoch-2 (Fig. 5f) compared to epoch-1 (Fig. 5c), resulting in fewer LLCs during epoch-2 (1 per year) than in epoch-1 (1.57 per year). Therefore, the southward shift in the equatorial winds and LLC decline in the BoB are predominantly associated with the PDO phases. Regardless of the ENSO phases, the frequency of LLC declined in epoch-2 (Table 1).

Several studies^42–44^ have questioned the reliability of the TC data in the pre-satellite era due to the changes in technology and analysis protocols. Most of these studies hint at an underestimation of the intensity of TCs in the pre-satellite era. After a detailed analysis of the NIO TC data^15^ from 1871 to 2010, it was found that the best-track data from the 1960s is reliable (see Supplementary Notes). The decline in the LLC frequency in the recent epoch is also apparent and more pronounced if the period of the study is limited to 1961 to 2010. The TC intensity data before 1980 depends on ship track density, polar-orbiting satellite intervals, and the density of the coastal observing stations. Given the uncertainty in the quality of the pre-satellite era TC intensity data, the results on the marginal increase in the strength of LLCs in the recent epoch must be interpreted with caution. Our analysis for this study is limited to the post-1950 period because of the larger uncertainties in TC data prior to 1950. Therefore, there is only one cycle of PDO, and any competing influence from the increased anthropogenic greenhouse gases resulting in increased surface temperature and changes in circulation cannot be eliminated. Despite these complexities, the evolution of PDO indices and the latitudinal position of zero absolute vorticity (*η* = 0) for the period 1900–2010 show a statistically significant correlation, indicating the robustness of this relationship (Supplementary Fig. 11). The mismatch between these two time series is more evident before 1920, when data are less reliable.

## Discussion

We conclude that the recent epoch (epoch-2, 1981–2010) has seen a remarkable decline in the post-monsoon LLC frequency over the north Indian Ocean in comparison with the earlier epoch (epoch-1, 1951–1980). This decline in LLC frequency (Fig. 1) cannot be attributed to an increasing SST and oceanic heat content and nearly unchanged mid-tropospheric humidity. The decline in LLC frequency in the recent epoch seems to be primarily caused by the reduced low-level vorticity (Figs. 2e, 3b) due to southward displacement in equatorial westerly winds and a slightly increasing vertical wind shear^45^ (Fig. 2d). The strong equatorial westerlies lead to the formation of cyclonic circulation on either side of the equator within the latitudinal belts of 5°N–11°N and 5°–11°S (Fig. 3d). This southward displacement of equatorial westerlies (Fig. 3d) in the recent epoch is strongly associated with the PDO (Fig. 4c, d, Supplementary Figs. 10, 11). Although the LLC frequency has decreased in epoch-2, once an LLC is formed, favorable thermodynamic conditions in the low-latitudes and north of 11°N lead to the strengthening of the cyclonic storms in recent decades^20,21,25^. However, the strengthening of LLCs in recent decades must be interpreted with caution due to the unreliability of the TC intensity data in the pre-satellite era.

The results present an interesting situation where remote influence by natural climate variability (PDO) causes fewer cyclones, but favorable local thermodynamic conditions due to global warming make them slightly stronger^21,46^. When this tug-of-war between the natural and anthropogenic forcing changes, and they begin to work synergistically, the risk of severe cyclones in the post-monsoon north Indian Ocean may be amplified. These results may guide planning and mitigating LLC-induced disaster in the Indian subcontinent. The models with poor PDO simulation, therefore, should be treated with caution when they are used for future projections of LLC over the north Indian Ocean.

## Methods

### Data sets

We use the ERA5 reanalysis data^47,48^ from 1951–2010 to understand the epochal variation of atmospheric parameters. Monthly mean ocean temperature data from the UK Met Office Hadley Centre^49^ is used to estimate TCHP. The Extended Reconstructed Sea Surface Temperature dataset, version 5 (ERSSTv5^50^) from the National Oceanic and Atmospheric Administration (NOAA) is used. The tracks and number of cyclones formed between 1951 and 2010 were obtained from the archives of the India Meteorological Department (IMD)^51^. Tracks for three types of cyclonic disturbances namely depressions (D), cyclonic storms (CS) and severe cyclonic storms (SCS) based on wind speed (Supplementary Table 1) are available in IMD’s Cyclone eAtlas^9^. In our analysis, we have studied the CS and SCS formed in different latitudinal and longitudinal belts of the Bay of Bengal. The basemap in Fig. 1 is based on the satellite image from the Blue Marble Next Generation, NASA’s Earth Observatory.

### Statistical significance test

To assess the statistical significance of the epochal changes in ocean-atmospheric variables, we performed the Student’s *t* test using the equation:

1 $$t=\frac{{\overline{x}}_{1}-{\overline{x}}_{2}}{\sqrt{\frac{{s}_{1}^{2}}{{n}_{1}}+\frac{{s}_{2}^{2}}{{n}_{2}}}}$$

where *n*_1_ and *n*_2_ are the sample sizes for the variable in two epochs, *x*_1_ and *x*_2_; *s*_1_ and *s*_2_ are the standard deviations of *x*_1_ and *x*_2_, respectively.

### Correlation and linear regression analysis

The method used to investigate the relationship between the PDO and the Indian Ocean SST/lower-tropospheric winds involved a linear regression analysis (equation (2)). Before regression, the PDO index and SST/wind data were preprocessed by applying an 11-year running mean to reduce short-term fluctuations and emphasize longer-term patterns. The linear regression analysis was performed for each grid point in the SST/wind dataset using the regCoef function available in NCAR Command Language (NCL). The regCoef function allows for the computation of the *t*-statistic, which can be used to test the significance of the regression results.

2 $$Y=a+bX$$

where *X* and *Y* represent the PDO index and SST/winds, respectively; a is the y-intercept of the regression line, indicating the value of Y when the PDO index (*X*) is zero; b is the slope of the regression line, representing the change in *Y* (Indian Ocean SST or winds) for unit change in the PDO index (*X*)

We assessed the correlation between the PDO index and cyclone frequency using the Pearson correlation coefficient:

3 $$r=\frac{\mathop{\sum }\nolimits_{i=1}^{n}({x}_{i}-\overline{x})({y}_{i}-\overline{y})}{\sqrt{\mathop{\sum }\nolimits_{i=1}^{n}{({x}_{i}-\overline{x})}^{2}}\sqrt{\mathop{\sum }\nolimits_{i=1}^{n}{({y}_{i}-\overline{y})}^{2}}}$$

where *n* is the sample size; *x*_*i*_ and *y*_*i*_ are individual data points in the datasets for the variables x and y (from i=1 to i=n); $\overline{x}$ and $\overline{y}$ are the mean values of the *x* and *y* variables, respectively.

### Oceanic Niño Index

To identify the positive (El Niño) and negative (La Niña) phases of the ENSO, we obtained the Oceanic Niño Index (ONI^52^) from the Climate Prediction Center (CPC), National Oceanic and Atmospheric Administration (NOAA, http://www.cpc.ncep.noaa.gov). The ONI is a widely accepted index based on a three-month running mean of sea surface temperature (SST) anomalies in the Niño 3.4 region (5°N-–5°S, 120°–170°W). A year is considered as a El Niño phase if the ONI is greater than or equal to +0.5 °C for a minimum of four consecutive overlapping seasons. Similarly, a La Niña phase was identified when the ONI index was less than or equal to −0.5 °C for a minimum of four consecutive overlapping seasons.

### Supplementary information

Supplementary Information

### Source data

Source Data

## Data Availability

All data used in this study are publicly available. The ERA5 reanalysis data are available to download from the https://www.ecmwf.int/en/forecasts/datasets/reanalysis-datasets/era5. The HadISST is publicly available at https://www.metoffice.gov.uk/hadobs/hadisst.The tracks and frequency of the north Indian Ocean cyclones used in this study are available in the archives of the India Meteorological Department (https://rsmcnewdelhi.imd.gov.in). EN4 ocean temperature dataset is available at https://www.metoffice.gov.uk/hadobs/en4/. PDO index is available at http://research.jisao.washington.edu/pdo/PDO.latest.txt. Source data are provided with this paper.

## Code availability

The codes for analysis, statistical computing and graphics are based on freeware environments, R (version 4.0.5, https://www.R-project.org/), NCAR Command Language (Version 6.6.2; 10.5065/D6WD3XH5), Ferret (version 7.5, http://ferret.pmel.noaa.gov/Ferret/) and CDO (version 1.9.2, 10.5281/zenodo.3539275). All codes used in this study are available from the corresponding author upon request.
