# Supplementary material for: Pacific decadal oscillation causes fewer near-equatorial cyclones in the North Indian Ocean
Source: Nat Commun. 2023 Aug 28;14:5099. doi: 10.1038/s41467-023-40642-x (PMC10462712; doi:10.1038/s41467-023-40642-x)
Supplement: Supplementary file 1 — Supplementary Information [file 41467_2023_40642_MOESM1_ESM.pdf]

Supplementary Information for "Pacific Decadal Oscillation Causes Fewer Near-Equatorial Cyclones in the North Indian Ocean"

Shinto Roose[1,2], R. S. Ajayamohan[1,3,*], Pallav Ray[4], Shang-Ping Xie[5], C. T. Sabeerali[1,6], M. Mohapatra[7], S. Taraphdar[1,8], K. Mohanakumar[9], and M. Rajeevan[10]

[1]Arabian Center for Climate and Environmental Sciences, New York University Abu Dhabi, Abu Dhabi, UAE
[2]Department of Civil Engineering, McGill University, Montreal, Canada
[3]Department of Meteorology, Abu Dhabi Polytechnic, Institute of Applied Technology, Abu Dhabi, UAE
[4]Meteorology, Florida Institute of Technology, Melbourne, FL, USA
[5]Scripps Institute of Oceanography, University of California San Diego, CA, USA
[6]Climate Research and Services, India Meteorological Department, Pune, India
[7]India Meteorological Department, New Delhi, India
[8]Atmospheric Science & Global Change Division, Pacific Northwest National Laboratory, WA, USA
[9]Advanced Centre for Atmospheric Radar Research, Cochin University of Science and Technology, India
[10]Ministry of Earth Sciences, New Delhi, India
*Ajaya.Mohan@nyu.edu

Supplementary Information

Supplementary Notes

Note on quality of data of Tropical Cyclones over the north Indian Ocean basin.

The technology and analysis protocols for tropical cyclone (TC) genesis and its intensity may have progressively evolved over time. Therefore, it is quite natural to expect some inconsistencies in the TC datasets. The published literature suggests that the decline in low-latitude cyclone (LLC) frequency is real, even though there may be uncertainty in the magnitude of the decline. On the other hand, the increase in the intensity of the LLCs from epoch-1 (1951-1980) to epoch-2 (1981-2010), although consistent with other recent studies, may have larger uncertainties due to various reasons. These issues related to the frequency and intensity of the LLCs in the north Indian Ocean (NIO) are discussed below.

TC Frequency.

1. The evolution of data collection and analysis protocols for TC genesis and intensity used by the India Meteorological Department (IMD) from 1877 to 2010 was highlighted by Mohapatra et al. (2012)[1]. During 1951-1960, there were 58 coastal surface observatories, 16 pilot balloon stations, and 6 Radiosonde stations (see Table 6 in Mohapatra et al (2012))[1]. A network of coastal stations near southern India, Andaman and Nicobar Islands, and Sri Lanka were established in 1952 (see Fig. 4 and Fig. 5 in Mohapatra et al (2010)[1]). The number of stations systematically increased after that. Based on the observational network, it was found that the genesis location, intensity, movement (track), and landfall can be best represented on the dataset from 1960 onwards. Therefore, if we define our epoch-1 as 1961-1980 and epoch-2 as 1981-2000, we still notice an epochal decline of LLCs from 38 to 18. Moreover, if we consider only the landfalling LLCs, the TC numbers have decreased from 26 to 14 during the same period. It is highly unlikely that the surface observational network will miss any of the landfalling storms from 1960 onwards. And even if some landfalling LLCs are missed, the chances of missing those LLCs are arguably larger in epoch-1 than in epoch-2. Therefore, the epochal decline in LLCs is also confirmed in the presence of better and denser observational networks from the 1960s.

2. The storm genesis positions before the satellite-era contain uncertainty, especially for the storm occurrence in the open oceans[2]. Veechi and Knutson (2011) suggested that an upward adjustment of hurricane counts may be needed during the pre-satellite era (before 1965) to account for likely "missed" TCs due to the sparse density of reporting ships in the North Atlantic. In the absence of any such study over the NIO, if we assume that this study would be qualitatively valid

for NIO as well, then there would be "missed" TCs in the pre-satellite era in the NIO also. This means that the epochal decline in LLC frequency in our study may have been underestimated. The ship observations were quite high during the pre-satellite era, and many ships were registered under IVOF (Indian Voluntary Observing Fleet; (see Section 5.4 in Mohapatra et al (2012)[1]). In our study, TC genesis over 5°N-11°N in the NIO is defined as LLC. Therefore, it is highly likely that even in the presence of inaccuracy in the reported genesis locations, the epochal decline in NIO LLC will persist. And lastly, the LLC tracks show that they generally move west-, north-, or northwest-ward (Fig. 1 of the paper). Therefore, the chance of an undetected LLC, later detected by an observational network north of 11°N, will not be classified as an LLC. This again shows that the number of LLCs in the pre-satellite era may have been underestimated. (On the other hand, the number of storms that form within 0°-5°N is negligible in the NIO. So an over count of LLCs due to storms formed south of 5°N and reaching 5°N and beyond is negligible).

3. There is often a question regarding whether the storms reported by the IMD suffered from a change in operational procedures that led to a change in classification. IMD added storm categories on the high wind side (Very Severe Cyclonic Storms and Super Cyclonic Storms) in 1974, and 1999 onwards based on the improvement in the observational network. The basic nomenclature remains the same, and there is no change in standard operating procedure for determining the location and intensity of TC. The IMD best track data takes into account the changes in the analysis protocols and has found that the data from 1961 is reliable[1]. Our conclusion regarding the decline in LLCs is also valid if we consider data from 1961.

4. Apart from the cyclone e-Atlas data, there are three more datasets available over the NIO during the analysis period. They are IBTrACS[3], IMD's Best Track data[1], and JTWC data[4]. The similar epochal decline is clearly evident in the IBTrACS, IBTrACS-IMD, and JTWC data as well, indicating the robustness of LLC decline in various datasets (see Table S4).

TC Intensity.

Any trend analysis of TC intensities remains problematic due to a host of issues in the NIO. For example, Kossin et al. (2013)[5] mentioned that there was no satellite coverage for the entire Arabian Sea before 1998 as well as the Bay of Bengal before 1981. Therefore, the storm intensity might have been biased due to the oblique view offered by adjacent satellites or subjective analysis by experts. They showed a large spatial gap in data (their Fig. 1) based on Meteosat and GEOS satellites. However, they did not consider polar-orbiting satellites that were in operational use by the IMD from the 1960s, and any system with a lifespan of more than 12 h could not be missed[1]. Indian geostationary satellites, INSAT series, were operational since 1983 and were used for TC monitoring over the Bay of Bengal and the Arabian Sea. Moreover, coastal observatories were augmented in the 1940s and 1950s, followed by Indian satellites in the 1960s (See Section 5 in Mohapatra et al (2010)[1]), which further helped in the detection of TCs and their intensity. Even with the above augmentation of observations, few studies[6] provided examples of a few NIO TCs that typically underestimated wind speeds and hence were treated as category-3 instead of category-4/5 TCs. Note that the number of LLCs in the Arabian Sea (AS) is very few (10 from 1951-2010). Hence, the data uncertainty in the AS[5] will not change any of our conclusions.

Genesis Potential Index (GPI) and relative role of factors that contribute to GPI.

We used the GPI (equation 1 and equation 2) to further understand the relative role of environmental parameters on epochal decline in LLC keeping in mind the known weaknesses in GPI. For example, the GPI typically can capture the seasonal cycle of TC frequency but cannot capture the interannual variability. The ability of the cyclogenesis indices to capture the seasonal cycle of TC numbers is not surprising, given that these indices are historically fitted on seasonal time scales. Also, the GPIs are typically developed for a larger geographical region or for global application. As a result, their performance over a small region (such as our LLC domain), that too in capturing the interannual variation in TC counts, is expected to be limited. For example, Menkes et al. (2012)[7] evaluated four cyclogenesis indices and found the following: (i) At the interannual timescale, none of the indices was able to reproduce the observed cyclogenesis, and (ii) most indices showed an equatorward bias in mean cyclogenesis, especially in the Northern Hemisphere where this bias can reach up to 5°. Therefore, GPI-based analysis over a region close to the equator is not reliable. As a result, we estimate the relative roles of the environmental parameters on TC genesis at a box (9°-13°N, 83°-95°E) that is further away from the equator than our LLC genesis domain (5°-11°N, 83°-95°E). Therefore, one may expect that the GPI may work better over this region compared to our LLC domain[7]. Also, in this chosen box, there was either decline or no change in LLC frequency (see Figure 1d of the manuscript). The results (Table 5) show that GPI decline is dominated by absolute vorticity (62.63%) and vertical wind shear (42.1%) with smaller contribution from relative humidity (17.69%). Similarly, when we consider another box (8°-13°N, 83°-95°E) that is also further away from the equator than our chosen LLC genesis domain (5°-11°N, 83°-95°E), the decline in GPI was contributed by absolute vorticity (66.02%) and vertical wind shear (49.99%). The results are similar when we consider other areas north of 7°N. Overall,

GPI-based analysis was able to differentiate between the factors that were conducive for cyclogenesis (maximum potential intensity and relative humidity) and the factor that were not conducive (vorticity and vertical wind shear) for cyclogenesis in epoch-2 compared to epoch-1 even over smaller regions away from the equator. Absolute vorticity seems to be a dominant factor with important contribution from the vertical wind shear that negatively impacted LLC genesis in epoch-2 compared to epoch-1. However, the vertical wind shear in ERA5 and NCEP-NCAR reanalysis show opposite epochal change (Figure S6). So, the influence of vertical wind shear on the epochal changes in LLC frequency may have large uncertainty. Also, our LLC genesis domain is close to the equator, and in the equatorial region, the number of storms is limited by small absolute vorticity even in the presence of other parameters that may be favorable for cyclogenesis.

Supplementary Equations.

Estimation of GPI.

GPI[8] is derived using ERA5 reanalysis data[9,10] from 1951-2010.

$$GPI = \underbrace{|10^5\ \eta|^{3/2}}_{Term1} \times \underbrace{(H/50)^3}_{Term2} \times \underbrace{(MPI/70)^3}_{Term3} \times \underbrace{(1+0.1\ V_{shear})^{-2}}_{Term4} \tag{1}$$

where η is the absolute vorticity at 850 hPa, H is the relative humidity at 600 hPa, MPI is the maximum potential intensity[11], V_{shear} is the vertical wind shear between 850 hPa and 200 hPa.

Estimation of relative contribution of four environmental parameters that constitute GPI

The relative contribution of each parameter to the change in GPI is estimated following the methodology outlined in Li et al. 2013[12].

$$\delta GPI = \alpha_1 \times \delta Term1 + \alpha_2 \times \delta Term2 + \alpha_3 \times \delta Term3 + \alpha_4 \times \delta Term4, \tag{2}$$

where,

$$\alpha_1 = \overline{Term2} \times \overline{Term3} \times \overline{Term4},$$

$$\alpha_2 = \overline{Term1} \times \overline{Term3} \times \overline{Term4},$$

$$\alpha_3 = \overline{Term1} \times \overline{Term2} \times \overline{Term4},$$

$$\alpha_4 = \overline{Term1} \times \overline{Term2} \times \overline{Term3},$$

In the above equations, a horizontal bar denotes the climatology, and δ represents the epochal difference in each term.

Supplementary Tables.

Supplementary Table 1 | Tropical cyclone classification criteria in the north Indian Ocean. Classification of cyclonic systems in the north Indian Ocean based on maximum sustained wind speed adopted by India Meteorological Department.

Tropical cyclone classification	Wind speed in knot
Depression (D)	17-27
Deep depression (DD)	28-33
Cyclonic storm (CS)	34-47
Severe cyclonic storm (SCS)	48-63
Very severe cyclonic storm (VSCS)	64-89
Extremely severe cyclonic storm (ESCS)	90-119
Super cyclonic storm (SupCS)	≥120

Supplementary Table 2 | Tropical cyclone (TC) genesis locations in the north Indian Ocean (NIO). The number of TCs in the low latitudes (5-11°N) of Bay of Bengal (BoB) (83-95°E) and NIO (60-100°E) based on IMD eAtlas dataset in the post-monsoon season during epoch-1 (1951-1980) and epoch-2 (1981-2010). TCs formed north of 11°N are also shown for the entire BoB and NIO.

Epoch	Number of LLCs (5-11°N)			TCs north of 11°N			Total BoB (83-95°E)	Total NIO (60-100°E)
	BoB (83-95°E)	Entire BoB (80-100°E)	NIO (60-100°E)	BoB (83-95°E)	Entire BoB (80-100°E)	NIO (60-100°E)		
Epoch-1	46	50	62	20	20	23	66	85
Epoch-2	26	29	35	21	27	35	47	70
Epoch-2 minus 1	-20	-21	-27	1	7	12	-19	-15

Supplementary Table 3 | low-latitude cyclone (LLC) frequency during pre-monsoon season. The number of LLCs (83°-95°E, 5°-11°N) during the pre-monsoon season (April-May) over the Bay of Bengal during epoch-1 (1951-1980) and epoch-2 (1981-2010) from IMD eAtlas (IBTrACS) dataset.

Epochs	Number of LLC
Epoch-1	9 (10)
Epoch-2	7 (7)

Supplementary Table 4 | low-latitude cyclone (LLC) frequency in the north Indian Ocean (NIO) from different datasets. The number of LLCs in the NIO basin based on IMD eAtlas/IBTrACS/JTWC datasets during the post-monsoon season[3,4,13] indicating the robustness of LLC decline in various datasets. The difference in LLC numbers is caused by the estimation of maximum sustained wind speeds (MSW) and TC categorization by various agencies. IBTracks-IMD represents counts of LLCs categorized as per the IMD criteria[13]. Note that IBTrACS does not provide wind speed information for the entire analysis period (1951-2010). Wind speed information from three agencies were substituted to check whether the IBTrACS matches IMD e-Atlas. DS824 winds were used for the period 1981-1990, USA agencies winds for 1981-1990 and RSMC (New Delhi) winds for 1990-2010, respectively. JTWC's intensity estimates are 5-10 Knots higher than IMD[14] leading to a much higher number of LLCs. For example, weak storms whose peak MSW speeds were just above the IMD cyclone threshold (34 Knots) for one or two 6-hourly periods, were reported by JTWC, but not by the IMD. Besides, JTWC data from 1945-1970 have not been reviewed or updated over the NIO.

Epochs	IMD e-Atlas	IBTrACS	IBTrACS-IMD	JTWC
Epoch-1	46	58	40	55
Epoch-2	26	33	23	26

Supplementary Table 5 | Relative contributions of tropical cyclogenesis parameters. Relative contributions of different parameters to epochal change in GPI[12,15] over the low latitudinal region (9°-13°N, 83°-95°E) in the Bay of Bengal during the post-monsoon season.

Term	Change ($\alpha \times \delta Term$)	Relative Contribution to $\delta GP1$
Absolute vorticity term	-0.2	+62.63%
Vertical wind shear term	-0.13	+42.1%
Relative Humidity term	-0.06	+17.69%
Potential intensity term	+0.07	-22.42%
δGPI=-0.31		

Supplementary Figures

.

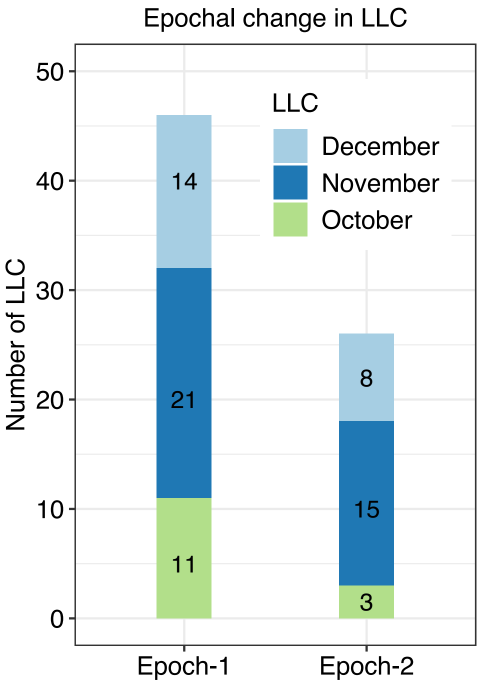

Supplementary Figure 1 | Epochal change in low-latitude cyclone (LLC) in the Bay of Bengal. The number of LLCs over the Bay of Bengal (83°-95°E, 5°-11°N) during post-monsoon season of epoch-1 and epoch-2.

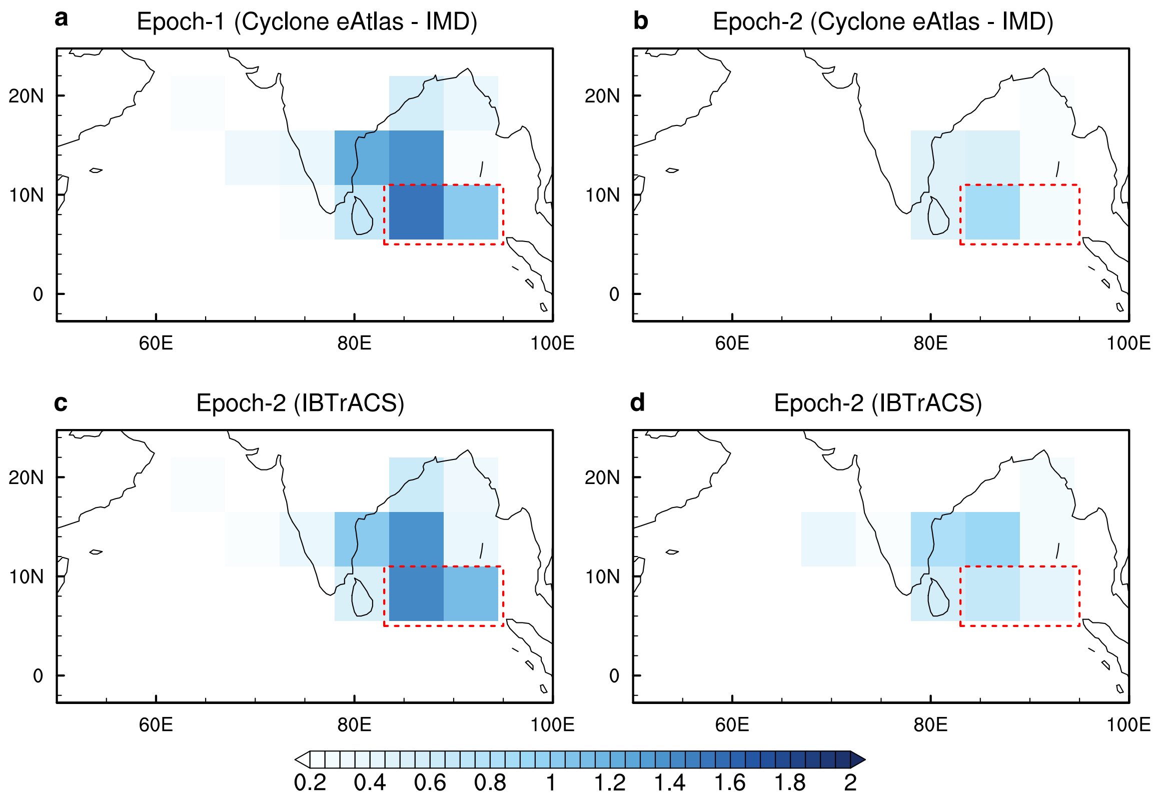

Supplementary Figure 2 | Tropical cyclone (TC) day density. TC day density per year (calculated by counting the number of days low-latitude cyclones (LLCs formed between 5°-11°N and 83°-95°E) spent over a 5.5°x5.5° grid box (from 0°-27.5°N and 45°-100°E) during each epoch using **a, b** IMD's Cyclone eAtlas and **c, d** IBTrACS datasets. The genesis location of the TC selected for this analysis is marked by the red dashed box.

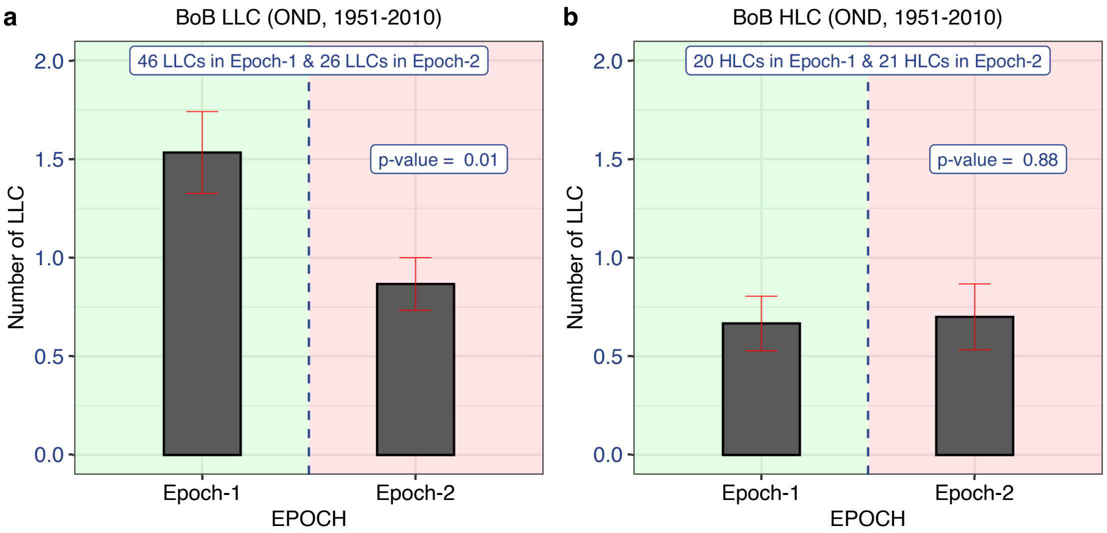

Supplementary Figure 3 | Epochal frequency of low- and high-latitude tropical cyclones. Epochal frequency of **a** LLCs (low-latitude cyclones formed in 83°-95°E, 5°-11°N) and **b** HLCs (high-latitude cyclones that formed north of 11°N and 83°-95°E). The standard error (SE, $standard\ deviation/\sqrt{sample\ size}$) is marked as a red bar.

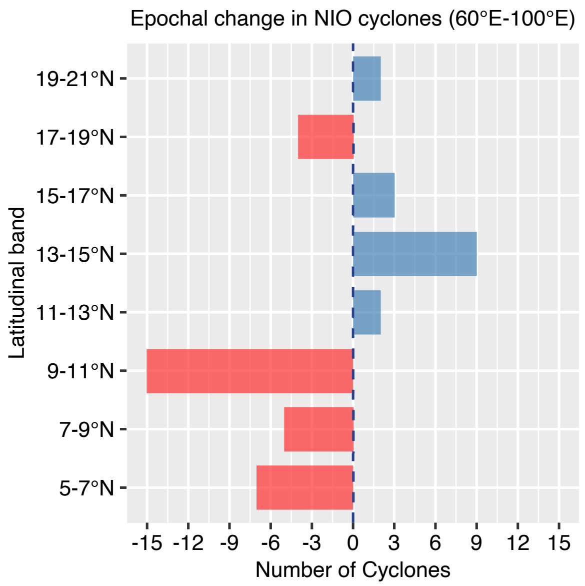

Supplementary Figure 4 | Latitudinal distribution of epochal change in tropical cyclone frequency. Latitudinal distribution of the epochal difference (epoch-2 minus epoch-1) in the number of cyclones in the north Indian Ocean (60°-100°E) during post-monsoon season

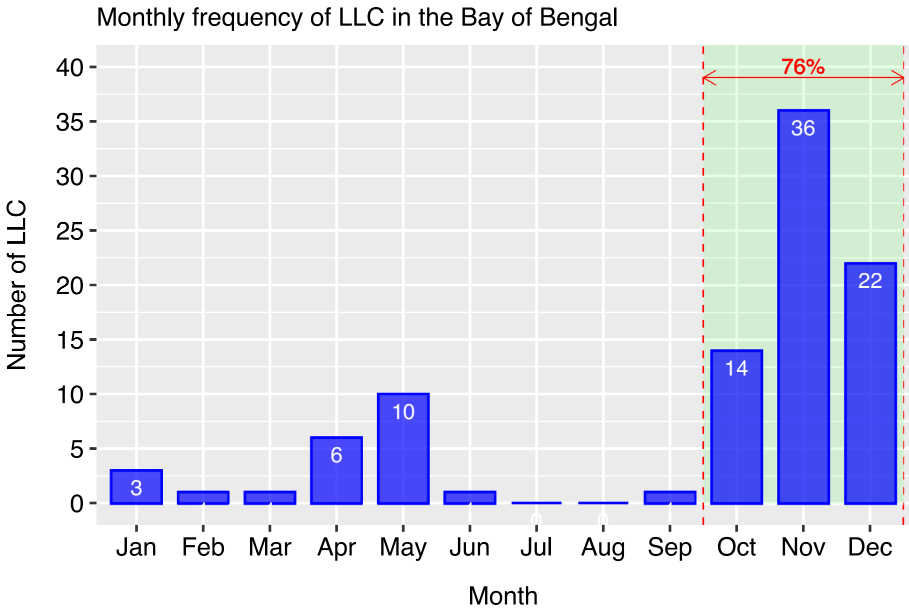

Supplementary Figure 5 | Monthly distribution of low-latitude cyclone (LLC) frequency. Monthly frequency of LLC in the Bay of Bengal (83°-95°E, 5°-11°N).

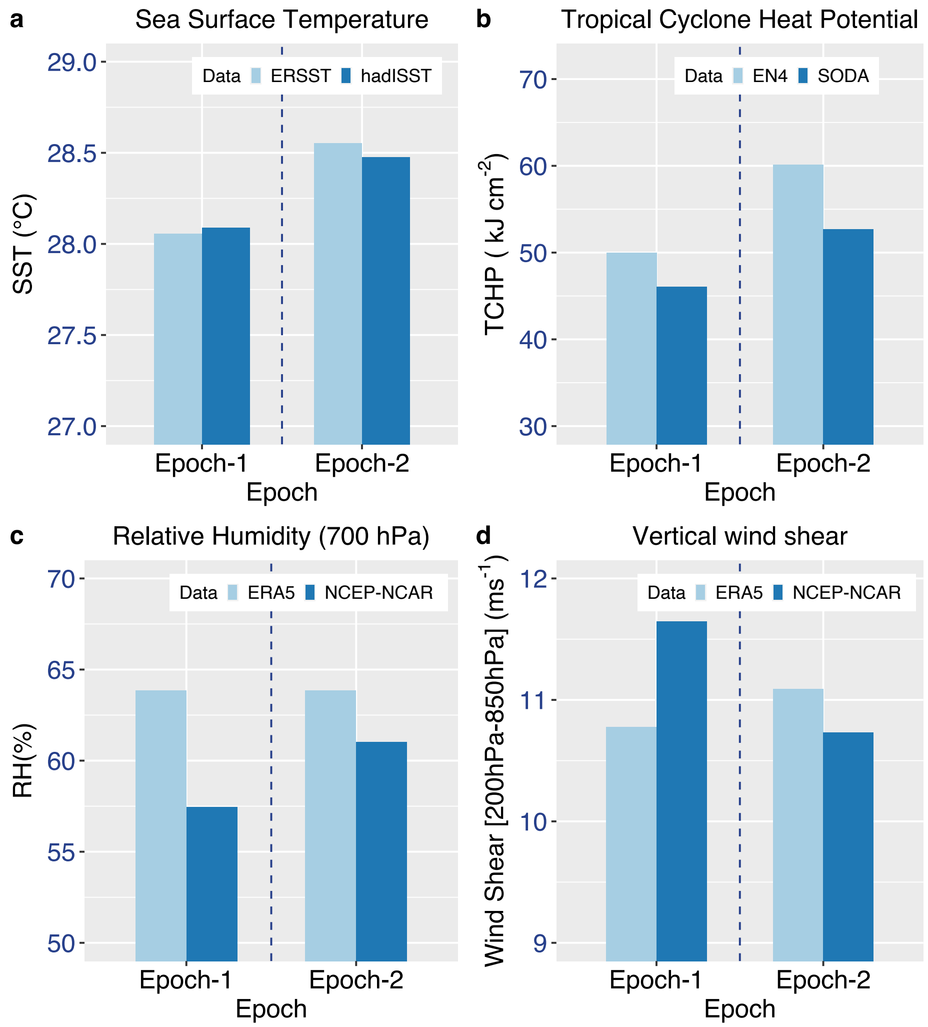

Supplementary Figure 6 | Epochal mean of tropical cyclone genesis parameters. Epochal mean of **a** sea surface temperature from ERSST[16] and HadISST[17] **b** tropical cyclone heat potential estimated from ocean temperature using EN4[18] and SODA[19], **c** mid-tropospheric (700 hPa) relative humidity, and **d** vertical wind shear between 850 and 200 hPa averaged over 83°-95°E, 5°-11°N during the post-monsoon season using different datasets. The relative humidity and verical wind shear are from ERA5[9,10] and NCEP-NCAR[20]. The vertical dashed lines separate the two epochs.

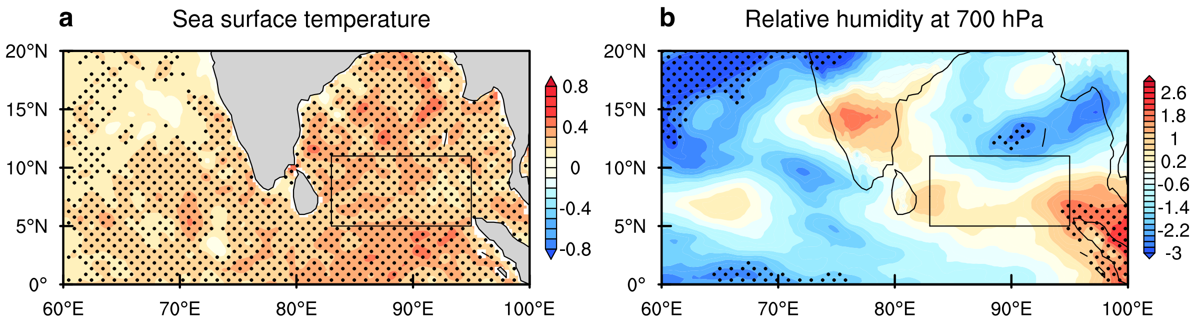

Supplementary Figure 7 | Epochal change in sea surface temperature (SST) and relative humidity. Epochal change in **a** sea surface temperature (°C, ERA5) and **b** mid-tropospheric (700 hPa) relative humidity (%, ERA5) during post-monsoon season. Stippling denotes areas where the significance exceeds 90% confidence level by a Student's *t*-test. The black rectangular area (83°-95°E, 5°-11°N) denotes the genesis location of low-latitude cyclones.

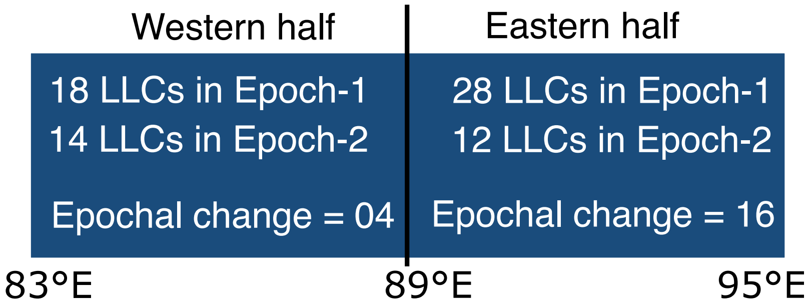

Supplementary Figure 8 | low-latitude cyclone (LLC) frequency in the eastern and western Bay of Bengal. Number of LLCs formed in epoch-1 (1951-1980) and epoch-2 (1981-2010) in the eastern half and western half of the LLC genesis domain (83° -95°E, 5°-11°N).

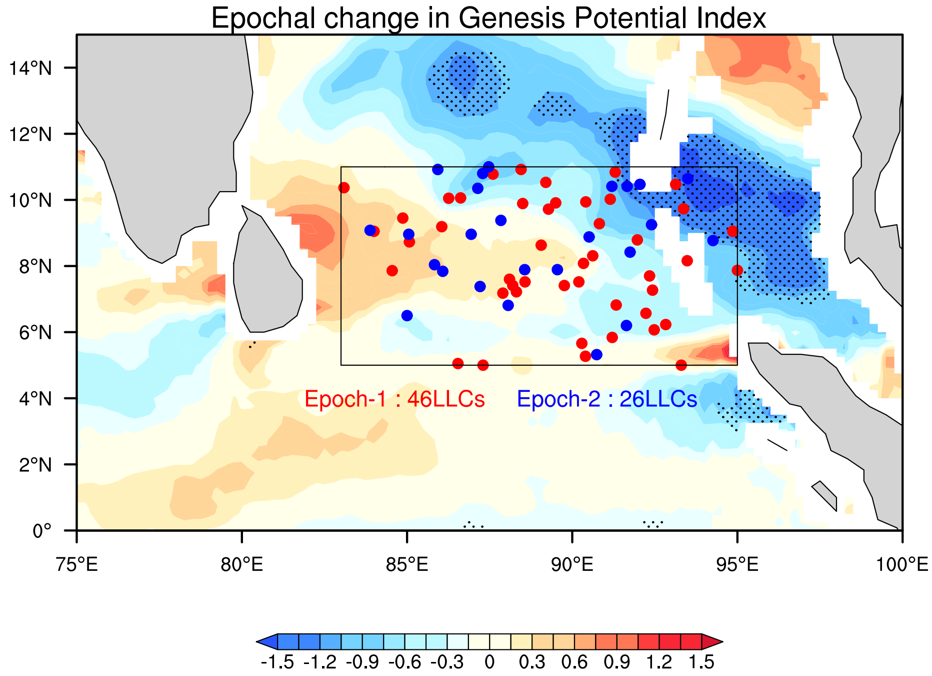

Supplementary Figure 9 | Epochal change in genesis potential index. Epochal change (epoch-2 minus epoch-1) in genesis potential index (GPI[8], dimensionless, Supplementary Equation 1)). Stippling denotes areas where the significance exceeds 90% confidence level by a Student's *t*-test. Genesis locations of low-latitude cyclones are marked as dots in red (epoch-1) and blue (epoch-2)

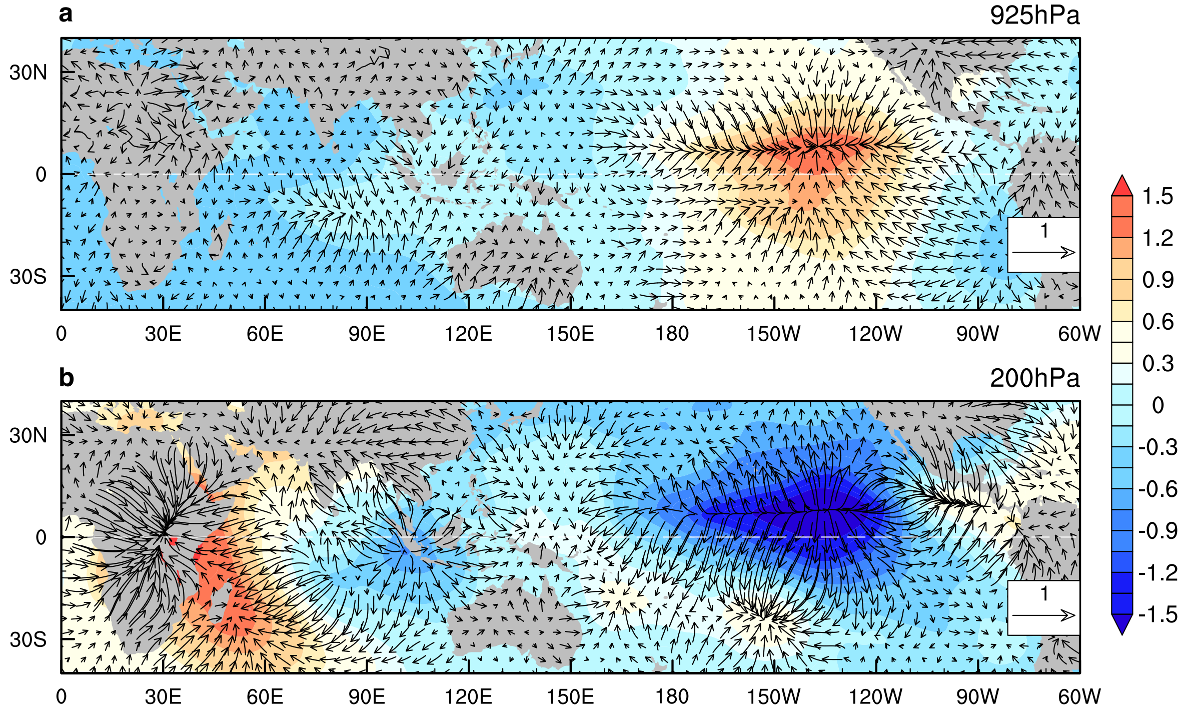

Supplementary Figure 10 | Epochal change in velocity potential and divergent winds. Epochal difference (1981-2010 minus 1951-1980) in velocity potential ($\times\ 10^6$ m^2 s^{-1}, shaded) and divergent winds (m s^{-1}, vector) at **a** 925 hPa and **b** 200 hPa during the post-monsoon season (ERA5).

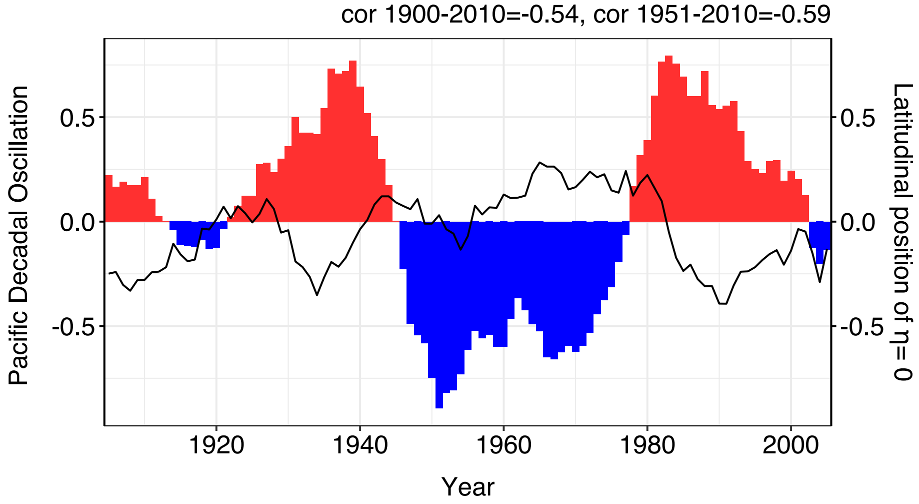

Supplementary Figure 11 | Relationship between Pacific Decadal Oscillation (PDO) and zero absolute vorticity. 11-year running mean of PDO index (shaded) and Latitudinal position of zero absolute vorticity at 850hPa (η=0) at 80°E during the post-monsoon season estimated from ERA-20C dataset[21]

Supplementary References.

1. Mohapatra, M., Bandyopadhyay, B. & Tyagi, A. Best track parameters of tropical cyclones over the north Indian Ocean: A review. *Nat. Hazards* **63**, 1285–1317, DOI: 10.1007/s11069-011-9935-0 (2012).
2. Vecchi, G. A. & Knutson, T. R. Estimating annual numbers of Atlantic hurricanes missing from the HURDAT database (1878–1965) using ship track density. *J. Clim.* **24**, 1736–1746, DOI: 10.1175/2010JCLI3810.1 (2011).
3. Knapp, K. R., Kruk, M. C., Levinson, D. H., Diamond, H. J. & Neumann, C. J. The international best track archive for climate stewardship (IBTrACS) unifying tropical cyclone data. *Bull. Amer. Meteorol. Soc.* **91**, 363–376, DOI: 10.1175/2009BAMS2755.1 (2010).
4. Chu, J.-H., Sampson, C. R., Levine, A. S. & Fukada, E. The joint typhoon warning center tropical cyclone best-tracks, 1945–2000. Tech. Rep., Joint Typhoon Warning Center, Naval Research Laboratory,Ref. NRL/MR/7540-02 (2002). Available at https://www.metoc.navy.mil/jtwc/products/best-tracks/tc-bt-report.html.
5. Kossin, J. P., Olander, T. L. & Knapp, K. R. Trend analysis with a new global record of tropical cyclone intensity. *J. Clim.* **26**, 9960–9976, DOI: 10.1175/JCLI-D-13-00262.1 (2013).
6. Landsea, C. W., Harper, B. A., Hoarau, K. & Knaff, J. A. Can we detect trends in extreme tropical cyclones? *Science* **313**, 452–454, DOI: 10.1126/science.1128448 (2006).
7. Menkes, C. E. *et al.* Comparison of tropical cyclogenesis indices on seasonal to interannual timescales. *Clim. Dynam.* **38**, 301–321, DOI: 10.1007/s00382-011-1126-x (2012).
8. Emanuel, K. & Nolan, D. S. Tropical cyclone activity and the global climate system. In *26th Conference on Hurricanes and Tropical Meteorology*, 240–241 (2004).
9. Hersbach, H. *et al.* The ERA5 global reanalysis. *Quart. J. Roy. Meteorol. Soc.* **146**, 1999–2049, DOI: 10.1002/qj.3803 (2020).
10. European Centre for Medium-Range Weather Forecasts. ERA5 back extension 1950-1978 (preliminary version), DOI: 10.5065/YBW7-YG52 (2020). Available at https://doi.org/10.5065/YBW7-YG52, Accessed 26 Nov 2020.
11. Gilford, D. M. pyPI (v1.3): tropical cyclone potential intensity calculations in python. *Geosci. Model. Dev.* **14**, 2351–2369, DOI: 10.5194/gmd-14-2351-2021 (2021).
12. Li, Z., Yu, W., Li, T., Murty, V. & Tangang, F. Bimodal character of cyclone climatology in the Bay of Bengal modulated by monsoon seasonal cycle. *J. Clim.* **26**, 1033–1046, DOI: 10.1175/JCLI-D-11-00627.1 (2013).
13. Cyclone-eAtlas. Tracks of cyclones and depressions over north Indian Ocean. Version 2.0, Cyclone Warning & Research Centre, India Meteorological Department, Chennai (2011). Available at http://www.rmcchennaieatlas.tn.nic.in.
14. Evan, A. T. & Camargo, S. J. A Climatology of Arabian Sea Cyclonic Storms. *J. Clim.* **24**, 140–158, DOI: 10.1175/2010JCLI3611.1 (2011).
15. Emanuel, K. & Nolan, D. S. Tropical cyclone activity and the global climate system. In *Extended Abstracts, 26th Conference on Hurricanes and Tropical Meteorology*, 240–241 (Amer. Meteor. Soc., 2004). Available at https://ams.confex.com/ams/26HURR/techprogram/paper_75463.htm.
16. Boyin, H. *et al.* NOAA Extended Reconstructed Sea Surface Temperature (ERSST), Version 5. Tech. Rep., NOAA National Centers for Environmental Information (2017). DOI: 10.7289/V5T72FNM.
17. Rayner, N. A. *et al.* Global analyses of sea surface temperature, sea ice, and night marine air temperature since the late nineteenth century. *J. Geophys. Res.* **108 (D14)**, DOI: 10.1029/2002JD002670 (2003).
18. Good, S. A., Martin, M. J. & Rayner, N. A. EN4: Quality controlled ocean temperature and salinity profiles and monthly objective analyses with uncertainty estimates. *J. Geophys. Res.* **118**, 6704–6716, DOI: 10.1002/2013JC009067 (2013).
19. Carton, J. A., Chepurin, G., Cao, X. & Giese, B. A simple ocean data assimilation analysis of the global upper ocean 1950-95 Part I: Methodology. *J. Phys. Ocean.* **30**, 294–309 (2000).
20. Kalnay, E. *et al.* The NCEP/NCAR 40-Year Reanalysis Project. *Bull. Amer. Meteorol. Soc.* **77**, 437–472, DOI: 10.1175/1520-0477(1996)077<0437:TNYRP>2.0.CO;2 (1996).
21. Poli, P. *et al.* ERA-20C: An atmospheric reanalysis of the twentieth century. *J. Clim.* **29**, 4083–4097, DOI: 10.1175/JCLI-D-15-0556.1 (2016).
